# Nobiletin Inhibits IL-1β-Induced Inflammation in Chondrocytes *via* Suppression of NF-κB Signaling and Attenuates Osteoarthritis in Mice

**DOI:** 10.3389/fphar.2019.00570

**Published:** 2019-05-31

**Authors:** Zeng Lin, Dengying Wu, Lipeng Huang, Chao Jiang, Tianlong Pan, Xiaodiao Kang, Jun Pan

**Affiliations:** Department of Orthopaedics, The Second Affiliated Hospital and Yuying Children’s Hospital of Wenzhou Medical University, Zhejiang, China

**Keywords:** nobiletin, osteoarthritis, interleukin-1β, chondrocyte, inflammation, NF-κB

## Abstract

Osteoarthritis (OA), a common degenerative joint disease, is principally characterized by inflammation and destruction of cartilage. Nobiletin, an extract of the peel of citrus fruits, is known to have anti-inflammatory properties. However, the mechanisms by which nobiletin plays a protective role in osteoarthritis (OA) are not completely understood. In the present study, we investigated the anti-inflammatory effects of nobiletin in the progression of OA in both *in vitro* and *in vivo* experiments. Mouse chondrocytes were pretreated with nobiletin (0, 10, 20, 40 μM) for 24 h and then incubated with IL-1β (10 ng/ml, 24 h) *in vitro*. The generation of PGE2 and NO was evaluated by the Griess reaction and ELISAs. The protein expression of inducible nitric oxide synthase, matrix metalloproteinase-3, matrix metalloproteinase-13, A disintegrin and metalloproteinase with thrombospondin motifs-5 (ADAMTS5), cyclooxygenase-2, collagen II, and aggrecan was analyzed by Western blotting. Immunofluorescence and Western blot analysis were used to detect nuclear factor-κB (NF-κB) signaling molecules. Induction of proinflammatory and catabolic mediators by IL-1β stimulation of mouse chondrocytes could be partially blocked by treatment with nobiletin or ammonium pyrrolidine dithiocarbamate (an NF-κB inhibitor). Furthermore, our results indicated that nobiletin exhibited a therapeutic effect through active inhibition of the NF-κB signaling pathway. In a mouse model of OA, injection of nobiletin (20 mg/kg) every 2 days for 8 weeks after surgery inhibited cartilage destruction and synovitis. Taken together, our findings suggest that nobiletin may be a potential therapeutic agent for the treatment of OA.

## Introduction

Osteoarthritis (OA) is a common disease that often causes physical limitations, disability, and mental stress, particularly in older people (French et al., 2015). OA is characterized by loss of joint function and degradation of cartilage, accompanied by joint pain (Aicher and Rolauffs, 2013; Thysen et al., 2015). There is no cure for osteoarthritis, as it is difficult to restore the cartilage once it is destroyed (Shen et al., 2012). In recent years, a large number of studies have demonstrated that trauma, obesity, inflammation, and age have a huge influence on the disease. It has been confirmed that a large number of inflammatory factors, such as interleukin-6 (IL-6), interleukin-1β (IL-1β), and tumor necrosis factor-α (TNF-α), perform an important role in the progression of OA (Haq et al., 2010; Johnson and Hunter, 2014). Many studies have revealed that IL-1β reduces the synthesis of collagen II, aggrecan, and other extracellular matrix (ECM) components by disrupting the metabolic balance of chondrocytes (Goldring et al., 1994). Matrix metalloproteinases regulate cartilage matrix and IL-1β can boost their generation in chondrocytes (Daheshia and Yao, 2008). Therefore, inhibition of the expression of IL-1β and IL-1β-induced inflammatory mediators may be a potential therapeutic route for attenuating the progression of OA. Recently, researchers have investigated natural compounds as potential therapeutic agents for human diseases (Lu et al., 2015). For example, many anti-OA compounds have been widely used against OA, such as sanguinarine, resveratrol, and matrine.

Orange peel is rich in a number of bioactive flavonoids including their methylated derivatives. Polymethoxyflavones (PMFs) are common methylated derivatives that have been used in traditional medicine (Sergeev et al., 2010). Nobiletin (5,6,7,8,3′,4′-hexamethoxyflavone), the major PMF in citrus fruits, particularly in the peel, has multiple biological functions, including being an antioxidant and having anti-inflammatory properties. It has been demonstrated to be an effective drug that has been used against many diseases, such as asthma, gastritis, and Alzheimer’s disease (Yang et al., 2017). Nobiletin ameliorates synovitis in collagen-induced arthritis by inhibiting the MAPK/NF-κB signaling pathway, as shown in previous research (Li et al., 2007). In addition, it has been demonstrated that nobiletin inhibits endotoxic shock in mice through the NF-κB pathway. Furthermore, nobiletin can inhibit the DNA-binding activity of NF-κB and ROS generation in LPS-activated RAW 264.7 cells (Choi et al., 2007). However, the mechanism of the anti-inflammatory effects of nobiletin in OA remains unclear. Therefore, we used *in vitro* and *in vivo* experimental models to investigate the anti-inflammatory properties of nobiletin and uncover the mechanisms involved in its influence on the pathogenesis of OA.

## Materials and Methods

### Reagents and Chemicals

Nobiletin (purity ≥ 98%), collagenase-II, and dimethyl sulfoxide (DMSO) were obtained from Solarbio (Beijing, China). Recombinant mouse IL-1β was purchased from Novoprotein (China). Primary antibodies directed against A disintegrin and metalloproteinase with thrombospondin motifs-5 (ADAMTS5), collagen II, and glyceraldehyde 3-phosphate dehydrogenase (GAPDH) were purchased from Abcam (Cambridge, MA, USA), while primary antibodies against iNOS, MMP-3, MMP-13, cyclooxygenase-2 (COX-2), p-IkBα, IkBα, p-p65, and p65 were obtained from ProteinTech (Wuhan, China). Cell-Counting Kit-8 (CCK-8) was purchased from Dojindo (Kumamoto, Japan). Fetal bovine serum (FBS), Dulbecco’s modified Eagle’s medium (DMEM)/F12, 0.25% trypsin ethylenediaminetetraacetic acid (trypsin–EDTA), and bovine serum albumin (BSA) were purchased from Healthcare Life Sciences (Hyclone; Logan, UT, USA). Griess reagent and Bicinchoninic acid radioimmunoprecipitation assay lysis buffer were purchased from Solarbio (Beijing, China). The NF-kB inhibitor pyrrolidine dithiocarbamate (PDTC) was obtained from Abcam (Cambridge, UK). Goat anti-mouse horseradish peroxidase conjugates and goat anti-rabbit were purchased from Jackson ImmunoResearch (West Grove, PA, USA). Griess reagent and PGE2 ELISA kits were obtained from Bio-Swamp Life Science (Shanghai, China).

### Primary Chondrocyte Isolation and Culture

Mice were sacrificed in accordance with ethical approval obtained from the Medical Ethical Committee of the Second Affiliated Hospital, Wenzhou Medical University, and following the guidelines of the Animal Care and Use Committee of Wenzhou Medical University. Articular cartilage was obtained from the knees and femoral heads of the mice. Firstly, the articular cartilage pieces were washed with PBS at least three times. They were then digested in a 5–9 ml aliquot of 0.2% type II collagenase in 0.2% trypsin–EDTA solution for 45 min and then incubated with 2 mg/ml (0.1%) collagenase II at 37°C for 4–5 h. The digested cartilage samples were then centrifuged at 1,000 rpm for 3 min at 37°C and the cell pellets seeded into 100 mm culture flasks following disposal of the supernatants. The cells were cultured in DMEM/F12 with 10% FBS and 1% antibiotics (penicillin/streptomycin) in an atmosphere containing 5% CO_2_ at 37°C. Nobiletin (40 μM) and PDTC (10 mM) were added to the culture media 2 h prior to treatment with IL-1β (10 ng/ml). The cells were passaged using 0.25% trypsin EDTA solution (Solarbio; Shanghai, China) when 80–90% confluent. Cells from passages 1 to 3 only were used in experiments to avoid changes in phenotype.

### Effect of Nobiletin on Chondrocyte Viability

Cell viability was determined using a CCK-8 kit according to the manufacturer’s instructions. In brief, P3 mouse chondrocytes were seeded into 96-well plates (5,000 cells/well) and incubated for 24 h. The cells were then treated with a concentration gradient (0, 10, 20, 40, 50, 100, and 200 μM) of nobiletin for either 24 h or 48 h. For the next 24 h, half the cells were incubated in IL-1β (10 ng/ml). Finally, 10 μl CCK-8 solution was added to each well and incubated for 2 h before measurement of optical density at 450 nm with a spectrophotometer (ThermoFisher).

### NO and PGE2 Measurements

Chondrocytes (3 × 10^5^ cells/ml) were seeded in 6-well plates and treated with nobiletin (10, 20, or 40 μM) 24 h prior to the addition of IL-1β (10 ng/ml). They were incubated for 24 h, and then the concentration of NO was measured using the Griess reaction. The optical density of each sample was measured at a wavelength of 543 nm. The concentration of PGE2 in each culture was measured by ELISA (R&D Systems, Minneapolis, MN USA) according to the manufacturer’s instructions.

### Immunofluorescence Analysis

Chondrocytes (3 × 10^5^ cells/ml) were seeded in 6-well plates and incubated either with or without nobiletin (40 μM) for 24 h and then with or without IL-1β (10 ng/ml) for 2 h. The cells were then washed three times in PBS prior to fixation with 4% paraformaldehyde for 15 min. After fixation, the cells were rinsed three times and then treated with 0.1% Triton X-100 for 15 min at room temperature. Chondrocytes were blocked with goat serum and then incubated overnight with p65 antibody (1:200) at 4°C. The cells were washed with PBS and incubated with fluorescein-conjugated goat anti-rabbit IgG antibody (1:400) for 1 h. Finally, the cell nuclei were stained with DAPI (Solarbio, Beijing, China) after washing three times with PBS.

### Animal Model of OA

Forty-five 10-week-old male C57BL/6 wild-type (WT) mice were purchased from the Animal Center of the Chinese Academy of Sciences, Shanghai. All experiments were conducted in accordance with the Animal Care and Use Committee of Wenzhou Medical University. The mouse osteoarthritis model was established as previously described (Vasheghani et al., 2015). Mice were randomly separated into three groups (*n* = 15 in each): a control group (sham-operated), OA group (OA), and OA treated with nobiletin group (nobiletin). The right joint of each mouse was modified by destabilization of the medial meniscus (DMM) in the OA and nobiletin groups by surgical transection, with the nobiletin group additionally receiving a 20 mg/kg intraperitoneal injection of nobiletin every 2 days. The OA group instead received an intraperitoneal injection of an inert vehicle (DMSO). The mice were maintained at a constant temperature of 20 ± 2°C, a relative humidity of 50 ± 10%, within a 12-h light/dark cycle. Eight weeks after surgery, the mice were sacrificed. Articular tissue was retained for further analysis.

### Histological Analysis

Briefly, the knee joint from each group was fixed in 4% paraformaldehyde for 24 h at 4°C and decalcified in 10% EDTA solution for 4 weeks. Frontal serial sections (6 μm thick) across the entire joint were obtained and stained with Safranin-O-Fast green stain (S-O) to evaluate cartilage destruction. The extent of cartilage degeneration in stained sections was assessed using light microscopy with the Osteoarthritis Research Society International (OARSI) scoring system, and synovitis scored, as previously described (Pritzker et al., 2006; Au et al., 2007). For immunohistochemistry, the sections embedded in paraffin were deparaffinized and rehydrated, and endogenous peroxidase was blocked by 3% hydrogen peroxide. The sections were incubated with 0.4% pepsin (Sangon Biotech, Shanghai, China) in 5 mM HCl at 37°C for 20 min for antigen retrieval. The sections were incubated with 5% bovine serum albumin for 30 min at room temperature, then the sections were incubated overnight at 4°C with primary antibodies for MMP-13, and finally with HRP-conjugated secondary antibody. Images were analyzed by Image-Pro Plus software, version 6.0 (Media Cybernetics, Rockville, MD, USA). Three sections from each group were used for quantitative analysis.

### Statistical Analysis

All experiments were performed at least three times. Data are presented as mean ± SD. Data were analyzed by one-way analysis of variance (ANOVA), followed by Tukey’s test for comparison between groups, using SPSS statistical software version 16.0. Nonparametric data (such as the OARSI score) were analyzed using a Kruskal-Wallis H test. *p* < 0.05 was considered significant.

## Results

### Potential Cytotoxicity of Nobiletin on Mouse Chondrocytes

**Figure 1:**
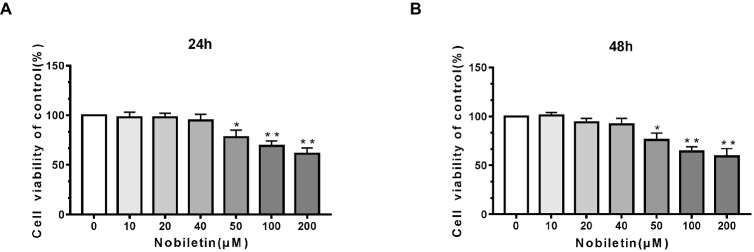
Effect of nobiletin on *in vitro* cell cytotoxicity. The cells were cultured with increasing concentrations of nobiletin (0, 10, 20, 40, 50, 100 or 200 μM) for 24 **(A)** and 48 h **(B)**. Cell viability was measured using a CCK-8 assay. Values represent mean ± SD of three independent experiments. Significant differences are indicated as *p < 0.05, **p < 0.01, compared to the control group.

To ascertain the cytotoxic effects of nobiletin on mouse chondrocytes, we performed a CCK-8 assay. The mouse cells were incubated with various concentrations of nobiletin (0, 10, 20, 40, 50, 100, and 200 μM) for 24 h. As shown in Figures 1A,B, nobiletin did not exhibit apparent cytotoxic effects on chondrocytes at concentrations of 0–40 μM, but it did at 50 μM and higher. The results of treatment for 48 h were similar. Therefore, nobiletin concentrations of 0, 10, 20 or 40 μM were used in all subsequent experiments.

### Effect of Nobiletin on PGE2, NO, TNF-α, and IL-6 Production in Mouse OA Chondrocytes

**Figure 2:**
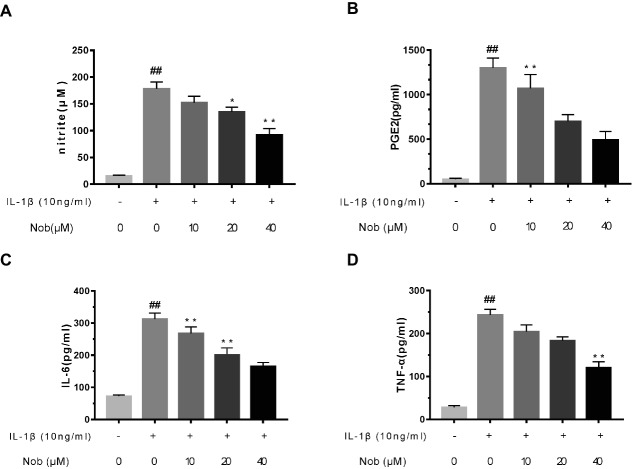
Effect of nobiletin on IL-1β-induced NO, PGE2, IL-6, and TNF-α production in mice OA chondrocytes. Chondrocytes were pretreated for 24 h with various concentrations of nobiletin (10, 20, or 40 μM) and then incubated for 24 h with or without IL-1β (10 ng/ml). Nitrite levels in the culture medium were assessed by Griess reaction **(A)**. Concentrations of PGE2, IL-6, and TNF-α were determined using ELISAs **(B–D)**. Values represent mean ± SD of three independent experiments. *p < 0.05, compared with control group. *p < 0.05 and **p < 0.01, compared with IL-1β group. ##*p* < 0.01, compared to the control group.

Firstly, the effects of nobiletin on IL-1β-induced PGE2 and NO production in chondrocytes were evaluated. Cells were pre-treated with different concentrations of nobiletin (0, 10, 20 and 40 μM, 24 h) and then stimulated with IL-1β (10 ng/ml, 24 h). The concentration of NO in the cell suspension was measured using the Griess reaction, while PGE2, TNF-α, and IL-6 levels were measured using ELISA kits. Levels of NO decreased significantly (*p* < 0.01) in combination with 10, 20, and 40 μM nobiletin (Figure 2A) compared to chondrocytes treated with just IL-1β, and this decrease was in a dose-dependent manner. Moreover, IL-1β-induction clearly increased the expression of IL-6, TNF-α, and PGE2 (*p* < 0.05) compared with the sham group. In addition, nobiletin also significantly decreased IL-6, PGE2, and TNF-α release in a dose-dependent manner (Figures 2B–D). The secretion of NO was in accordance with these results.

### Effects of Nobiletin on Cyclooxygenase-2, INOS, MMP-3, MMP-13, and ADAMTS5 Expression in Chondrocytes

**Figure 3:**
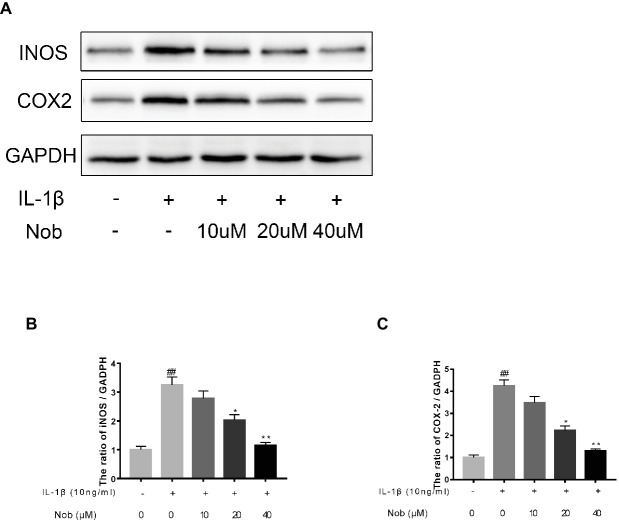
Effects of nobiletin on INOS and COX-2 expression in chondrocytes. Cells were pretreated with various concentrations of nobiletin (0, 10, 20, or 40 μM, 24 h) before stimulation with IL-1β (10 ng/ml, 24 h). Expression of INOS and COX-2 was assessed by Western blot analysis **(A–C)**. Values represent mean ± SD of three independent experiments. Significant differences are indicated as *p < 0.05, compared with control group. *p < 0.05 and **p < 0.01, compared with IL-1β group. ##*p* < 0.01, compared to the control group.

**Figure 4:**
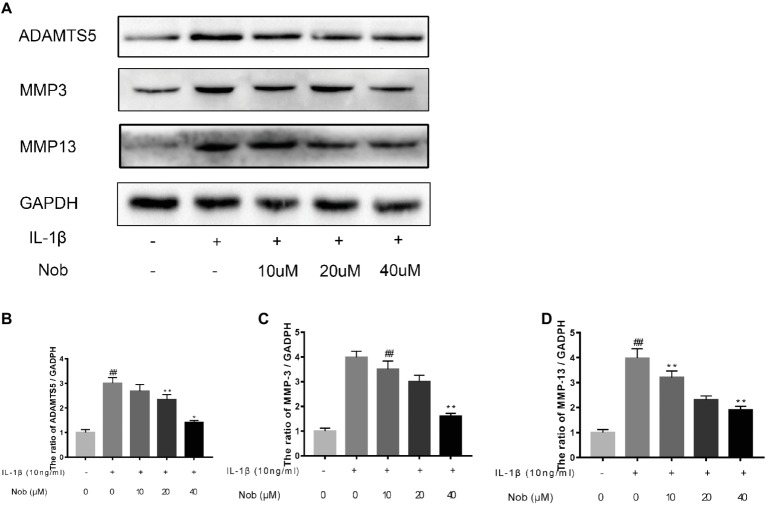
Effects of nobiletin on MMP-3, MMP-13, and ADAMTS-5 expression in chondrocytes. Cells were pretreated with various concentrations of nobiletin (0, 10, 20, or 40 μM, 24 h) prior to stimulation with IL-1β (10 ng/ml, 24 h). Expression of MMP-3, MMP-13, and ADAMTS-5 was evaluated by Western blot analysis **(A–D)**. Values represent mean ± SD of three independent experiments. Significant differences are indicated as *p < 0.05, compared with control group. *p < 0.05 and **p < 0.01, compared with IL-1β group. ##*p* < 0.01, compared to the control group.

We investigated the expression of COX-2, INOS, MMP-3, MMP-13, and ADAMTS5 in IL-1β-stimulated cells by western blotting. Western blot analysis demonstrated that inflammatory mediators (COX-2, INOS) (Figures 3A–C) and the synthesis of catabolic factors (MMP-3, MMP-13, ADAMTS5) (Figures 4A–D) were significantly upregulated in IL-1β-induced chondrocytes (10 ng/ml) for 24 h (*p* < 0.05). Nobiletin suppressed the expression of these mediators stimulated with IL-1β in a dose-dependent manner.

### Effect of Nobiletin on Aggrecan and Collagen II Expression in Mouse Chondrocytes

**Figure 5:**
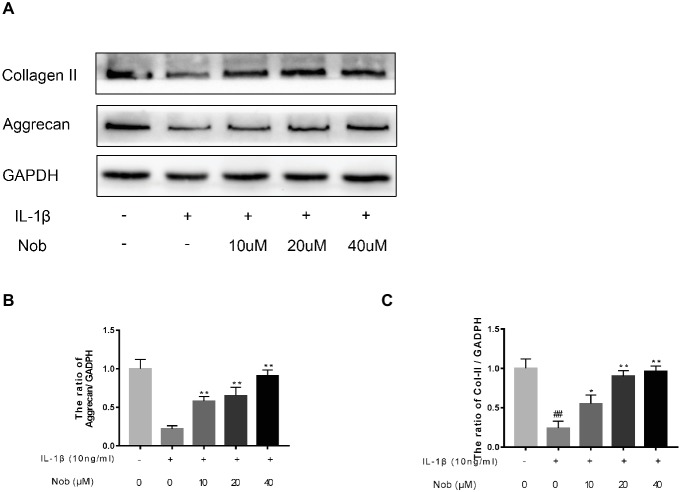
Effects of nobiletin on collagen II and aggrecan expression in mouse chondrocytes. Cells were pretreated with various concentrations of nobiletin (0, 10, 20, and 40 μM, 24 h) before stimulation with IL-1β (10 ng/ml, 24 h). The expression of collagen II and aggrecan was assessed by Western blot analysis **(A–C)**. Values represent mean ± SD of three independent experiments. Significant differences are indicated as *p < 0.05 and **p < 0.01, compared with control group. *p < 0.05 and **p < 0.01, compared with IL-1β group. ##*p* < 0.01, compared to the control group.

We examined the influence of nobiletin on IL-1β-induced collagen II and aggrecan expression in mouse chondrocytes. As shown in Figures 5A–C, protein generation of aggrecan and collagen II in the cells was distinctly downregulated after IL-1β stimulation compared with the control group (*p* < 0.05). In contrast, the decreased expression of aggrecan and collagen induced by IL-1β was reversed by nobiletin pretreatment in a dose-dependent manner.

### Effect of Nobiletin on NF-κB Signaling in Mouse Chondrocytes

**Figure 6:**
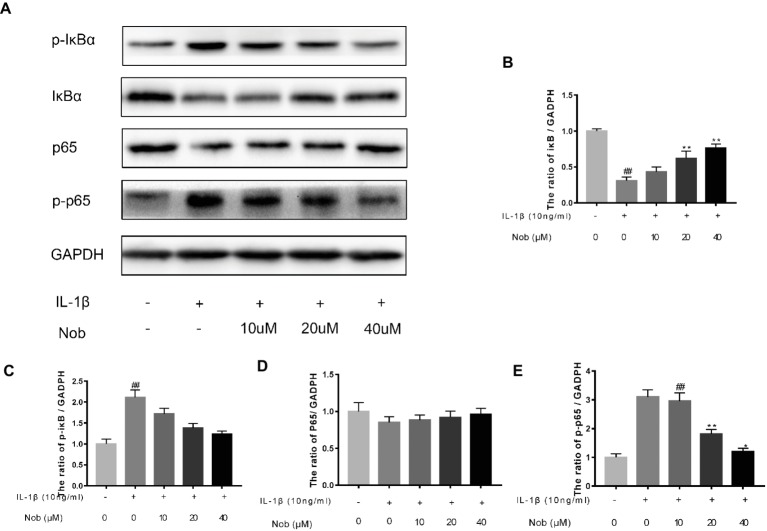
Effects of nobiletin on IL-1β-induced NF-κB activation in mouse OA chondrocytes. Chondrocytes were pretreated for 24 h with various concentrations of nobiletin (10, 20, or 40 μM), followed by incubation for 24 h with or without IL-1β (10 ng/ml). Protein expression levels of p65, p-p65, IκB, and p-IκB were determined by Western blot and quantification analysis **(A–E)**. Values represent mean ± SD of three independent experiments. ^##^ *p* < 0.05, compared with control group. *p < 0.05 and **p < 0.01, compared with IL-1β group.

**Figure 7:**
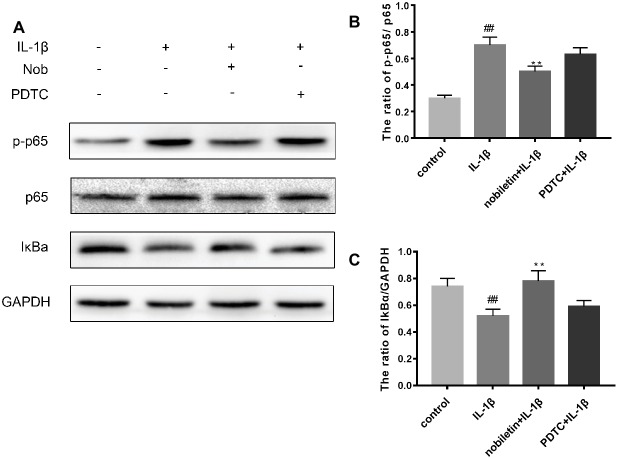
Effects of PBS, IL-1β, nobiletin with IL-1β, and PDTC with IL-1β on the NF-kB signaling pathway. **(A–C)** IL-1β significantly increased levels of p-p65 and decreased concentrations of IkBa, an effect which could be partially reversed through the addition of nobiletin or by treatment with PDTC. Values represent mean ± SD. ^##^ *p* < 0.01, compared to the control group. **p < 0.01.

We used Western blot analysis to evaluate the effect of nobiletin on nuclear factor-κB (NF-κB) signaling. The phosphorylation of NF-κB p65, IKKα/β, and IκBα was clearly increased in mouse chondrocytes by IL-1β induction (Figures 6A–E). Furthermore, IκBα levels became significantly degraded in chondrocytes after IL-1β stimulation. Conversely, nobiletin notably suppressed the IL-1β-induced phosphorylation of p65, IKKα/β, and IκBα and degradation of IκBα. To ascertain whether the NF-κB signaling pathway performs an important role in the negative consequences of IL-1β induction of chondrocytes, they were treated with PDTC, an NF-kB inhibitor. These experiments clearly demonstrated that PDTC partially inhibited the phosphorylation of NF-κB p65 and IκBα after treatment with IL-1β (Figure 7).

### Effect of Nobiletin on NF-κB p65 Nuclear Translocation in IL-1β-Induced Mouse OA Chondrocytes

**Figure 8:**
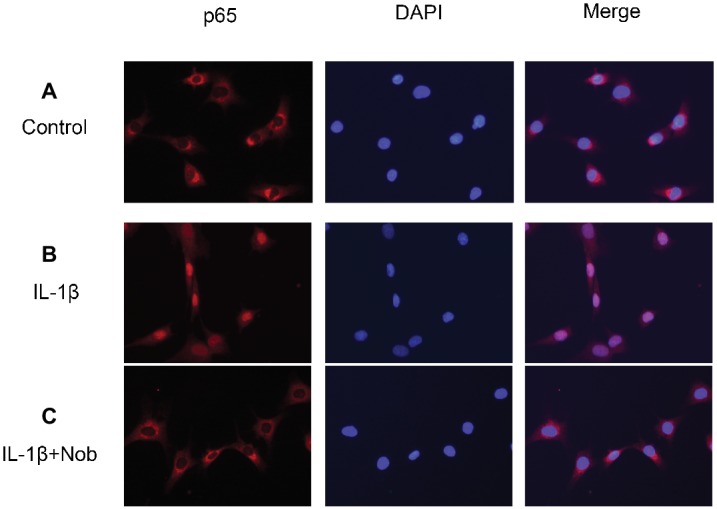
Immunofluorescence staining of NF-κB p65 protein in chondrocytes. Chondrocytes were untreated **(A)**, treated with IL-1β alone **(B)**, or treated with IL-1β and nobiletin (40 μM) **(C)**. In the untreated group, p65 was mainly restricted to the cytoplasm **(A)**. In the IL-1β alone group, p65 was translocated to the nucleus **(B)**, a phenomenon inhibited by pretreatment with nobiletin **(C)**. All experiments were repeated three times.

We wished to detect the effect of nobiletin on the nuclear translocation of NF-κB p65 in IL-1β-induced mouse chondrocytes. To accomplish this, we performed immunofluorescence microscopy. Cells were incubated with IL-1β (10 ng/ml) alone for 3 h or with IL-1β (10 ng/ml) and nobiletin (40 μM) for 24 h. Most p65 that was detected in the control group had accumulated in the cytoplasm, while in the IL-1β-treated group more was observed in the nucleus (Figure 8). However, nobiletin reversed the translocation of the p65 subunit of NF-κB to the nucleus. The above results illustrate that the action of nobiletin is in accordance with its inhibitory effect on NF-κB as observed by Western blot analysis.

### Nobiletin Suppresses the Degradation of Cartilage in a Mouse Model of OA

**Figure 9:**
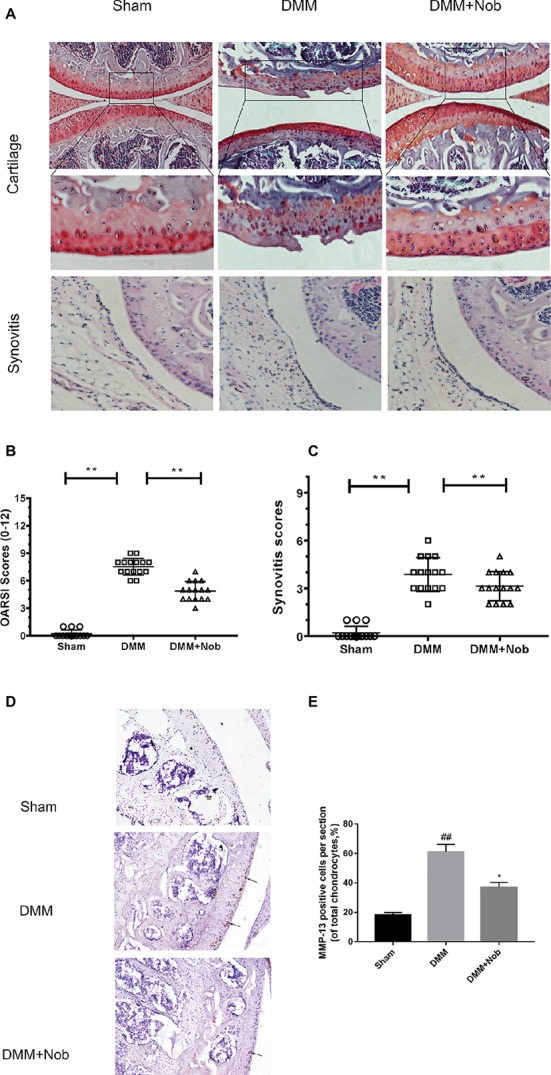
Nobiletin suppressed degradation of cartilage in a mouse model of OA. Safranin O staining was used to assess histomorphometric differences between the sham, OA, and OA + nobiletin groups. Mice received intraperitoneal injections of nobiletin (20 mg/kg) or an inert vehicle (DMSO) in the 8th week after surgery. Histological analysis of OA was evaluated by Safranin O staining **(A)** and hematoxylin and eosin (H&E) staining for synovitis, Osteoarthritis Research Society International (OARSI) scores **(B)**, and synovitis scores **(C)**. Immunohistochemistry of MMP-13 was assessed to the effect of nobiletin on cartilage matrix degradation in mice OA models (black arrows indicate the examples of MMP-13 positivity). Quantification of MMP-13-positive cells in cartilage samples **(D,E)**. Significant differences are indicated as *p < 0.05, compared with control group. *p < 0.05 and **p < 0.01, compared with OA group, *n* = 15. ##*p* < 0.01, compared to the control group.

To investigate whether nobiletin has the ability to prevent the induction or progression of osteoarthritis *in vivo*, a mouse model of OA was established. The model used the established DMM procedure, followed by treatment with nobiletin (20 mg/kg) every 2 days for 8 weeks. S-O staining was used with subsequent grading using OARSI scores to evaluate the severity of OA. As demonstrated in Figure 9A, the S-O staining shows that the surface of the articular cartilage of the OA group showed greater signs of destruction and devastating cartilage erosion, apparent hypocellularity, and vast proteoglycan loss in the OA group compared to the sham control group. However, the degradation of the cartilage matrix in the nobiletin group was intermediate between the OA and control groups and apparently had the effect of increasing the thickness of the articular cartilage and restoring any that had been destroyed. The OARSI scores (Figure 9B) accorded with the S-O staining results described above, demonstrating a clear difference in the nobiletin treatment group in the DMM mouse model compared with the OA group, with OARSI scores in the OA group that were clearly higher than those of the nobiletin group. Furthermore, the observed synovitis was significantly inhibited by nobiletin (Figure 9C) compared with OA group samples. Taken together, these results suggest that nobiletin is able to prevent the progression of osteoarthritis in a mouse model of OA. To further investigate the effect of nobiletin on ECM homeostasis *in vivo*, we performed immunohistochemical staining for MMP-13, a major collagenolytic enzyme involved in OA (Figures 9D,E). In the DMM group, marked MMP-13 positivity in the ECM and around chondrocytes was detected. In contrast, MMP-13 expression was significantly decreased by nobiletin.

## Discussion

Osteoarthritis, a chronic degenerative joint disease, is characterized by progressive loss of cartilage matrix and destruction of articular cartilage. With the passage of time, the subchondral bone thickens combined with the production of various inflammatory mediators in the synovium. Lastly, degeneration of the ligaments and menisci in the knee lead to hypertrophy of the joint capsule (Loeser, 2009). As a result, anti-inflammatory drugs are generally used to abate the progress of osteoarthritis, including steroids and nonsteroidal anti-inflammatory drugs. However, these only relieve articular pain and swelling, with overuse leading to deleterious side effects (Altman, 2009). Therefore, an effective and safe drug, which not only relieves clinical symptoms of OA but also slows its progression, is required. In recent years, researchers have shown interest in fruit-derived compounds as drugs for the treatment of OA, owing to their anti-inflammatory effects and lack of harmful side effects (Chen et al., 2017). Nobiletin, a major component in citrus fruits, is known for its pharmacological properties. It exhibits numerous bioactive effects including being anti-diabetic, suppressing cancer, being neurotrophic, preventing damage caused by oxidative stress, and performing a protective role in inflammatory diseases (Li et al., 2006; Choi et al., 2015).

In this study, we investigated whether nobiletin could inhibit IL-1β-induced secretion of inflammatory factors from chondrocytes and ameliorate the progression of OA in a model of OA in mice. In previous studies, inflammatory cytokines have been demonstrated as a pivotal factor in the progression of OA (Marks and Donaldson, 2005). IL-1β is the most important inflammatory cytokine and is frequently used in OA research. Chondrocytes induced by IL-1β generate COX-2 and iNOs, which results in the plentiful production of PGE2 and NO (Koch et al., 1989). iNOS is an important member of the nitric oxide synthase (NOS) family of enzymes and is capable of synthesizing NO (Sasaki et al., 1998; Clancy et al., 2001; Li et al., 2010). In addition, COX-2 is a critical mediator in OA that can generate an additional significant factor, PGE2. These inflammatory factors impact both bone resorption and joint pain (Hardy et al., 2002). Accumulated quantities of NO and PGE2 can induce cell to synthesize and release matrix metalloproteinase-13 (MMP-13), inhibiting the expression of type II collagen, ultimately leading to chondrocyte apoptosis (Brinckerhoff and Matrisian, 2002; Klein and Bischoff, 2011). In addition, recent studies have demonstrated that inhibiting the expression of inflammatory factors such as PGE2 and NO delays the degeneration of articular cartilage (Cheleschi et al., 2015). In the present study, we found that nobiletin suppressed PGE2 and COX-2 production in chondrocytes induced with IL-1β. These findings indicate that nobiletin reduces the progression of OA by blocking the generation of those inflammatory factors. MMPs are members of the principal family of proteolytic enzymes participating in the destruction of cartilage and disrupting the equilibrium between synthesis and degradation of extracellular matrix components (ECM) such as aggrecan and collagen II in OA (Cui et al., 2017). Levels of MMP-3 and MMP-13 are significantly increased in OA. Previous studies have reported that the levels of MMP-13 increase markedly as OA progresses (Goldring and Goldring, 2004). Consequently, we believe that a potential therapy for OA is the targeting of MMPs (Hu et al., 2005). In previous experiments, nobiletin reduced the production of MMP-3 and MMP-13 in IL-1β-stimulated cells. Thus, nobiletin may protect cartilage from degradation by OA through the suppression of MMP production, and so it may be a good potential candidate for curing OA. Among factors that are elevated as OA progresses, the ADAMTS family of proteins has been shown to clearly influence the degradation of cartilage, especially ADAMTS5. In a previous study, it was shown that ADAMTS5 cleaved aggrecan in the early stages of OA (Zhang et al., 2015). Subsequent experiments reported that inhibition of ADAMTS5 activity using siRNA reduced the loss of aggrecan in human OA cartilage explants, suggesting that it may be a potential therapeutic target for the treatment of OA.

Many components constitute cartilage matrix, of which aggrecan and collagen II are in the greatest concentration. They are essential for regulating the balance between catabolism and metabolism. Under normal circumstances, chondrocytes synthesize and secrete collagen II and aggrecan, thereby preventing mechanical damage (Wilusz et al., 2014). When cartilage destruction occurs, chondrocytes are exposed to nonphysiological loads causing large numbers of chondrocytes to apoptose. At the same time, aggrecan and collagen II undergo considerable degradation due to the secretion of high concentrations of proteolytic enzymes. These conditions accelerate the progression of OA (Lee et al., 2016).

The results in this study demonstrate that nobiletin can inhibit the decrease in collagen II and aggrecan levels in IL-1β-induced mouse chondrocytes. The mechanism of this inhibition due to nobiletin is possibly the suppression of proteolytic enzyme production, such as IL-1β-induced MMPs. In previous studies, researchers have demonstrated that the NF-κB signaling pathway regulates inflammation as OA progresses (Marcu et al., 2010).

In a resting state, NF-κB becomes inactive when combined with its inhibitory protein, IκB-α (Rigoglou and Papavassiliou, 2013). When stimulated by inflammatory factors such as IL-1β, inactive NF-κB becomes active and translocates from the cytoplasm to the nucleus. Ultimately, phosphorylation of p65 upregulates the expression of a large number of inflammatory factors, including COX-2, iNOS, PGE2, NO, MMPs, and ADAMTS, resulting in disruption of the balance of anabolic and catabolic function, and cartilage inflammation (Liacini et al., 2002). Furthermore, it has also been demonstrated that the use of NF-κB inhibitors can alleviate the joint swelling of collagen-induced arthritis in mice (Barnes and Karin, 1997; Rigoglou and Papavassiliou, 2013). Hence, in this study, we conducted a thorough investigation of whether nobiletin exerts its anti-inflammatory effects through the NF-κB signaling pathway in mouse chondrocytes. We found that phosphorylation of p65 and IκBα was clearly increased after induction by IL-1β. However, treatment with nobiletin caused a significant inhibition of phosphorylation levels in a dose-dependent manner. The NF-κB pathway plays a crucial role in the pathogenesis process of OA. To investigate whether this pathway was involved in the IL-1β-induced negative effect on chondrocytes, we used an NF-κB inhibitor, PDTC. In the present study, we found that PDTC partially blocked the IL-1β-induced NF-κB activation. Through the use of another technique (immunofluorescence staining), we detected that nobiletin suppressed the activation of NF-κB p65 translocation to the nucleus compared to stimulation with IL-1β. Taken together, the above results suggest that the mechanism of nobiletin’s ability to reduce the expression of inflammatory mediators may be the inhibition of the phosphorylation of NF-κB p65.

OA is a prevalent joint disease that involves many complicated mechanisms including cartilage degradation, synovial inflammation, and subchondral sclerosis. We established a mouse model of OA in this study to perform a deep investigation of the anti-inflammatory effects of nobiletin *in vivo*. In previous studies, DMM has been confirmed as a ubiquitous animal model of OA. Histological staining demonstrated that nobiletin dramatically inhibits the progression of OA in mice. Moreover, the OARSI grade was lower in mice treated with nobiletin. Through all these experiments, we have demonstrated the value of nobiletin as a treatment for OA. Nevertheless, we did not investigate the NF-κB signaling pathway in OA mice. Future studies exploring the effect of nobiletin on activation of the NF-κB pathway *in vivo* are required.

## Conclusions

In conclusion, we demonstrated that nobiletin can inhibit inflammatory conditions induced by IL-1β stimulation, including a reduction in the expression of ADAMTS5, MMP-3, MMP-13, INOS, and COX-2 in mouse OA chondrocytes. Moreover, the NF-κB signaling pathway may have performed an important role in this process. Our experiments confirmed that nobiletin can perform a protective role in cell culture in addition to an OA mouse model. Taken together, these findings indicate that nobiletin may have therapeutic implications for treating OA.

## Ethics Statement

This study was carried out in accordance with the recommendations of “Guide for the Care and Use of Laboratory Animals of the National Institutes of Health.” The protocol was approved by the “the Animal Care and Use Committee of Wenzhou Medical University.”

## Author Contributions

JP, ZL, and DW carried out the concept, design, definition of intellectual content, literature search, data acquisition, data analysis, and manuscript preparation. LH, CJ, TP, and XK provided assistance for data acquisition, data analysis, and statistical analysis. ZL and DW carried out literature search, data acquisition, and manuscript editing. JP performed the manuscript review. All authors have read and approved the content of the manuscript.

### Conflict of Interest Statement

The authors declare that the research was conducted in the absence of any commercial or financial relationships that could be construed as a potential conflict of interest.
